# A Study of the Comparison Between Preoperative Ultrasonography Findings With Intraoperative Observations in Laparoscopic Cholecystectomy

**DOI:** 10.7759/cureus.76159

**Published:** 2024-12-21

**Authors:** Amit Girme, Vernika Gupta

**Affiliations:** 1 General Surgery, Dr. Dnyandeo Yashwantrao Patil Medical College, Hospital and Research Centre, Dr. Dnyandeo Yashwantrao Patil Vidyapeeth (Deemed to be University), Pune, IND

**Keywords:** cholelithiasis, gall stone disease (gsd), intraoperative observations, laparoscopic cholecystectomy, preoperative ultrasonography

## Abstract

Aim: This study aims to evaluate the accuracy of ultrasonography (US) by comparing preoperative ultrasonographic findings with intraoperative observations during laparoscopic cholecystectomy (LC).

Materials and methods: An observational analytical study was conducted at a tertiary hospital in Pune over two years and included 98 patients aged 20-80 with symptomatic cholelithiasis confirmed by US. Preoperative parameters assessed included gallstone number, gallbladder volume, wall thickness, and pericholecystic fluid. These were compared to intraoperative findings during LC. Data were analyzed using SPSS Statistics for Windows, Version 16 (Released 2007; SPSS Inc., Chicago, Ill), with sensitivity, specificity, positive predictive value (PPV), and negative predictive value (NPV) calculated for the US parameters.

Results: The mean patient age was 44.14±15.06 years, with a female predominance (70%). US showed high sensitivity (78.13%) but low specificity (35.29%) for detecting the number of stones, and showed high specificity (100%) but low sensitivity (21%) in the assessment of gallbladder wall thickness. For detecting gallbladder distension, US had a sensitivity of 69.7% and a specificity of 11.1%, while it achieved perfect sensitivity and NPV (100%) for evaluation of pericholecystic fluid.

Conclusions: Preoperative US provides valuable insights for surgical planning, with high sensitivity and specificity for gallbladder wall thickness and pericholecystic fluid. However, its accuracy in detecting the number of stones and gallbladder distension is moderate. US can be considered a useful investigation for diagnosing cholelithiasis. It may be used to predict the fate of LC, but further studies need to be carried out to confirm its surgical importance.

## Introduction

Global health surveys reveal that approximately 14.2 million women and 6.3 million men are affected by cholelithiasis (gallstone disease or GSD), with an estimated annual incidence of 1.39 per 100 individuals [1]. Of these, one to three percent become symptomatic and require surgical intervention [1]. In India, GSD has a notable prevalence, occurring in four percent of the population [2]. The disease is more frequently observed in women and older populations, and is influenced by genetic, age-related, gender, and racial factors [3,4]. Patients with cholelithiasis often experience a dyspeptic symptom complex which includes right hypochondriac colicky pain and/or epigastric pain with bloating, belching, regurgitation, and nausea. Untreated, it can lead to severe complications like acute cholecystitis, choledocholithiasis, gallbladder perforation, pancreatitis, cholangitis, and Mirizzi syndrome [5].

Abdominal ultrasonography (US) is the first investigation for preoperative assessment since it lacks ionizing radiation and is non-invasive, widely available, cost-effective, and can evaluate adjacent organs [6,7]. It has been used for detecting GSD with a sensitivity of 84% and a specificity of 99% [8].

Laparoscopic cholecystectomy (LC) is considered the gold standard treatment for cholelithiasis [9]. Certain factors such as gallbladder (GB) wall thickness and pericholecystic fluid, when observed during the procedure, can complicate the surgery or necessitate its conversion into an open procedure, negating the benefits of a minimally-invasive approach. These factors can be assessed preoperatively using a US to predict the potential outcomes of LC [10].

This study compares preoperative ultrasonographic findings with intraoperative observations in LC. Its primary objective is to evaluate the accuracy of US as a diagnostic tool for planning LC, thereby enhancing preparedness and decision-making in patient care.

## Materials and methods

The study was conducted at the Department of General Surgery at Dr. D.Y. Patil Medical College, Hospital and Research Centre in Pune. It followed a cross-sectional analytical design and was conducted from 1st August 2022 to 31st July 2024. The sample consisted of 98 individuals selected by convenience sampling. Participants included symptomatic patients, aged between 20 and 80 years, diagnosed with cholelithiasis via US and who underwent LS. All participants provided written and informed consent for elective LC. The study excluded patients younger than 20 or older than 80 years, those with complicated gallbladder stone disease, patients unfit for laparoscopic surgeries based on pre-anesthetic checkups, and those who did not consent to LC.

The Institutional Ethics Committee approved the study (IESC/PGS/2022/79). Patients’ consent was obtained after they had been fully informed about the study. Participants were assured of the confidentiality of their reports.

The ultrasonographic parameters evaluated included the number of gallstones (GS; single/multiple), GB volume (distended >50 cc, non-distended 30-50 cc, contracted <30 cc), GB wall thickness (normal <4 mm, thickened >4 mm), and the presence or absence of pericholecystic fluid. Patients then underwent LC, during which intraoperative findings of the same parameters were recorded. Postoperatively, the specimen was examined to confirm these findings.

Data was collected in Microsoft Excel (Microsoft Corp., Redmond, WA, US) and analyzed using SPSS Statistics for Windows, Version 16 (Released 2007; SPSS Inc., Chicago, Ill). Qualitative data were presented as frequencies, while quantitative data were presented as mean ± standard deviation (SD). A Chi-Square test was used to compare the variables. The sensitivity, specificity, positive predictive value (PPV), and negative predictive value (NPV) of US were analyzed when evaluating the variables.

The study's conclusions were addressed in light of the materials, study design, and findings from other relevant studies. These findings were used to draw conclusions and make recommendations.

## Results

The mean age of patients with symptomatic cholelithiasis in this study was 44.14±15.06 years and it predominantly affected the middle-aged population. The gender distribution among participants showed a significant predominance of female patients, who made up 70% of the patient population, while male patients accounted for the rest. Among the observed symptoms, the most commonly reported one was 'dyspeptic symptoms,' affecting 88 patients, followed by colicky pain and nausea.

Table 1 presents a summary of the number of abnormalities of interest detected in the preoperative US divided by the total number of scans performed.

**Table 1:** A summary of the number of abnormalities of interest detected by the preoperative ultrasounds and during laparoscopic observations

Variables	Ultrasonographic findings (n=98)	Intraoperative findings (n=98)
Number of stones		
Single	26	34
Multiple	72	64
Gallbladder wall thickness		
Normal	4	19
Thickened	94	79
Gallbladder distension		
Distended	89	70
Non-distended	7	20
Contracted	2	8
Pericholecystic fluid		
Present	1	17
Absent	97	81

Table 2 represents the analysis of sensitivity, specificity, PPV, NPV, and accuracy of the preoperative US in evaluating variables with intraoperative findings during LC. US showed a moderate sensitivity (78.13%) but low specificity (35.29%) for detecting the number of gallstones, while it was highly specific (100%) for identifying GB wall thickness but with low sensitivity (21.05%). For pericholecystic fluid, US achieved perfect sensitivity (100%) and a high negative predictive value (100%), making it reliable for ruling out the condition.

**Table 2:** Analysis of sensitivity, specificity, PPV, NPV, and accuracy of preoperative ultrasonography in evaluating parameters with intraoperative findings. GB, gallbladder; PPV, positive predictive value; NPV, negative predictive value

Variables	Sensitivity	Specificity	PPV	NPV	Accuracy
Number of stones (single/multiple)	78.13%	35.29%	69.44%	46.15%	63.27%
GB wall thickness (thickened/normal)	21.05%	100.00%	100.00%	84.04%	84.69%
GB distension (contracted/distended/non-distended)					
Contracted	0%	91.70%	0%	97.80%	89.80%
Distended	69.70%	11.10%	88.60%	3.60%	64.30%
Non-distended	NA	NA	NA	NA	NA
Pericholecystic fluid (present/absent)	100%	83.51%	5.88%	100%	83.67%

**Figure 1:**
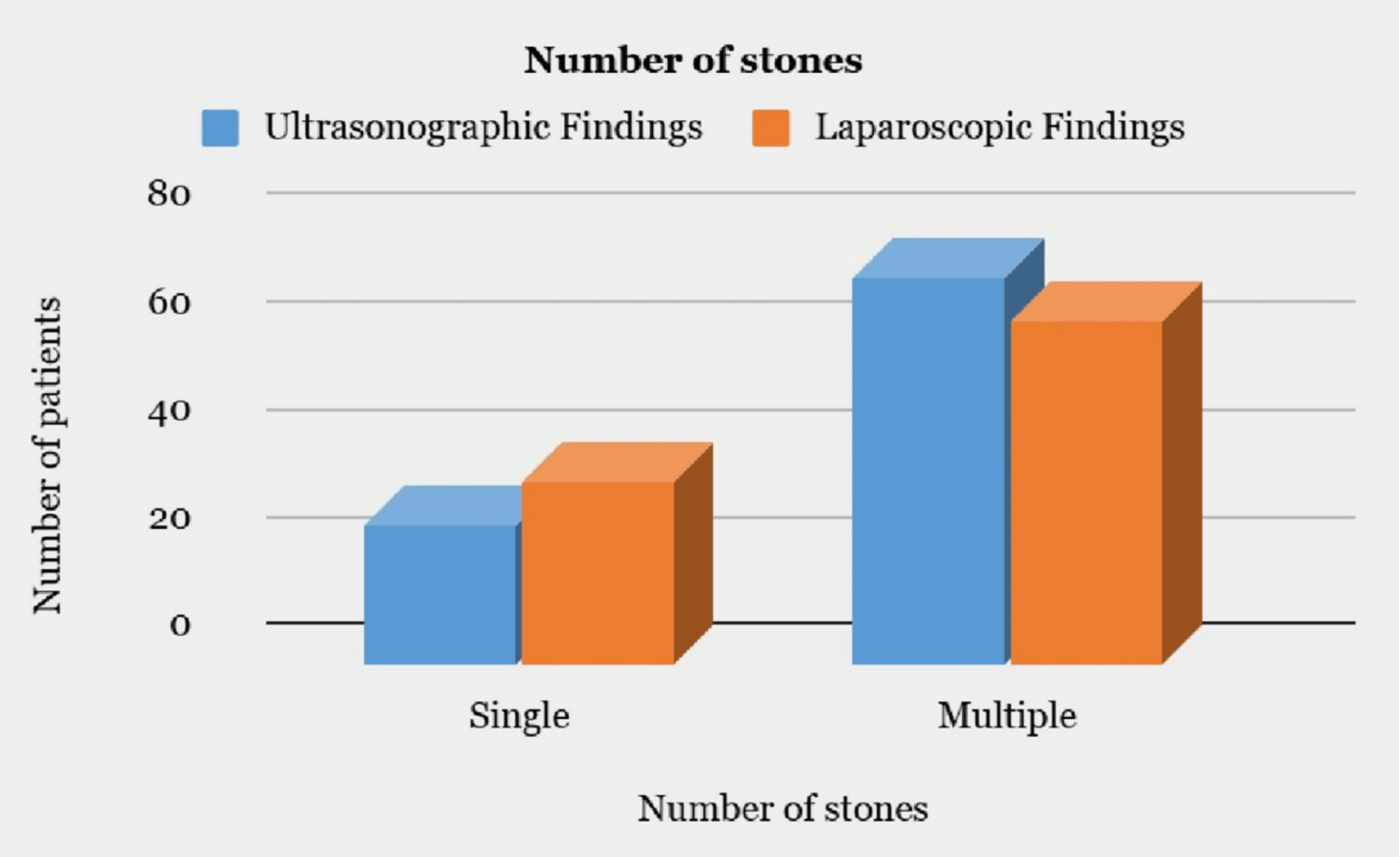
Comparison of ultrasonographic and laparoscopic findings with respect to number of stones

**Figure 2:**
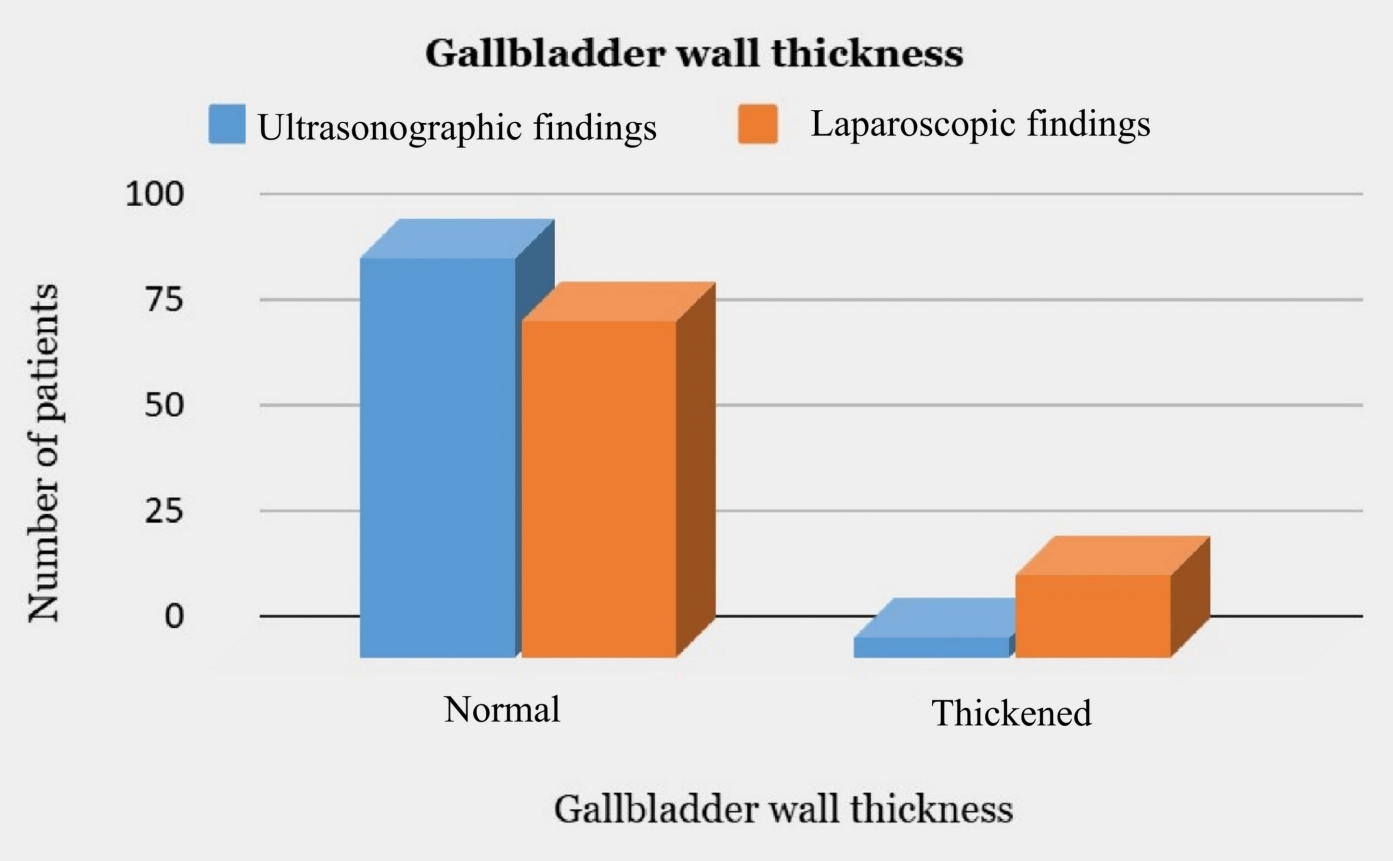
Comparison of ultrasonographic and laparoscopic findings with respect to gallbladder wall thickness

**Figure 3:**
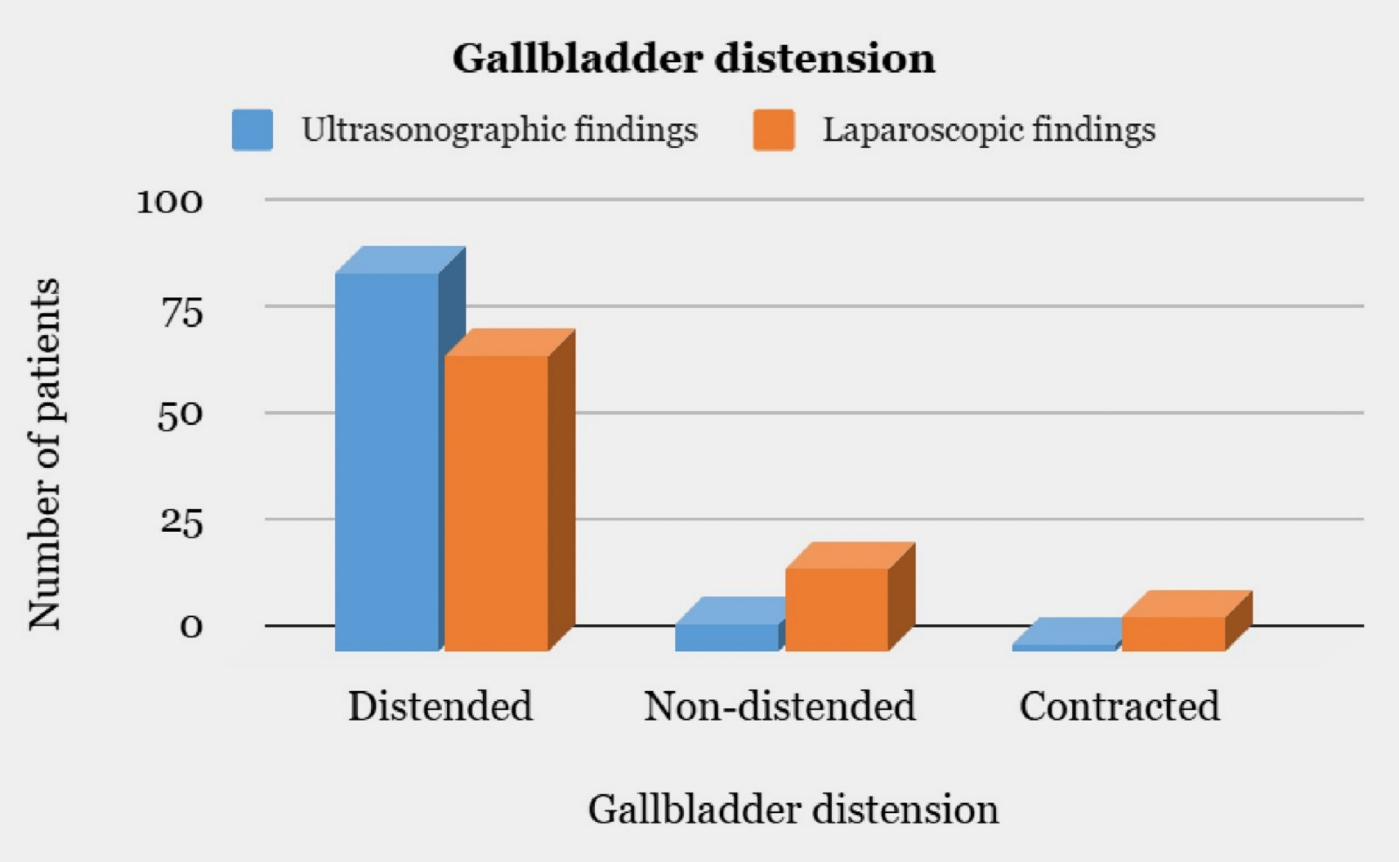
Comparison of ultrasonographic and laparoscopic findings with respect to gallbladder distension

**Figure 4:**
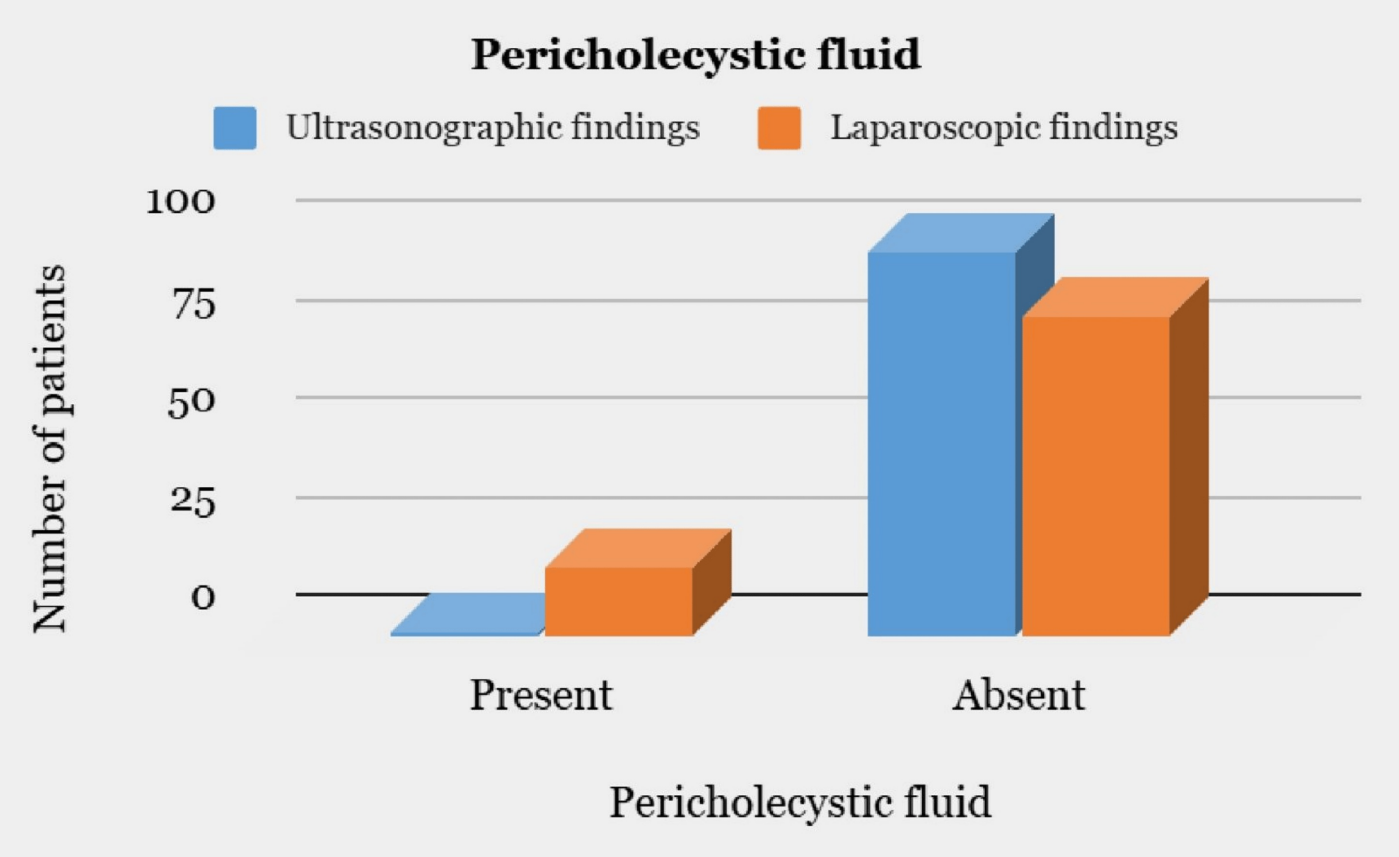
Comparison of ultrasonographic and laparoscopic findings with respect to pericholecystic fluid

The bar graphs (Figures 1-4) compare ultrasonographic and laparoscopic findings concerning the number of stones, GB wall thickness, GB distension, and pericholecystic fluid. Among the observed findings, US revealed 26 patients with a single stone and 72 patients with multiple stones. On the contrary, out of 98 patients, 34 were found to have a single stone intraoperatively, while 64 patients had multiple stones (Figure 1).

Among the observed findings, US revealed only 4 patients with a thickened GB wall while 94 patients had a normal report. Intraoperatively, 19 patients were found to have a thickened GB wall while 79 patients had normal GB wall thickness (Figure 2).

According to US, 89 patients had a distended GB, 7 had a non-distended GB and 2 patients had a contracted GB. During the LC, 70 patients had a distended GB, 20 had non-distended GB, and 8 patients had a contracted GB (Figure 3).

As per the assessment by US, only one patient had pericholecystic fluid while 97 patients did not have pericholecystic fluid. Intraoperatively, during LC, 17 patients were found to have pericholecystic fluid while 81 patients had no pericholecystic fluid (Figure 4).

For the number of stones, US showed relatively high sensitivity but low specificity, highlighting its limitations in accurately identifying true negatives. PPV and NPV indicated moderate reliability for the positive and negative results, respectively. Overall, the test's accuracy was moderate in assessing the number of stone. For GB wall thickness, the test performed well in confirming true negatives but showed limited sensitivity in identifying true positives. The metrics for GB wall distension, along with their 95% confidence intervals, provide insights into the accuracy and reliability of this test across different diagnostic outcomes. When applied for pericholecystic fluid, US achieved perfect sensitivity and NPV. However, the PPV was notably low at 5.88%, indicating its limited ability to correctly identify true positives.

## Discussion

This study involved 98 patients diagnosed with cholelithiasis via ultrasound who underwent LC at our institution. It aimed to explore the correlation between preoperative evaluations and intraoperative findings.

Each symptomatic patient underwent an abdominal US to confirm GSD diagnosis. The US identified gallstones and assessed their number and size. The correlation between preoperative ultrasound findings and intraoperative discoveries regarding gallstone numbers remains ambiguous. Research indicates that clusters of stones appearing clumped on US may manifest as a single large stone or multiple smaller stones during surgery.

In our study, US demonstrated a high sensitivity (78.13%) but a low specificity (35.29%) in detecting gallstones, suggesting a tendency for false positive results that necessitate further investigation and intervention to confirm diagnoses. Al-Timimi et al. reported an almost complete agreement (Kappa statistic=0.944) between US and the operative findings [11]. The present study had an accuracy of 63.27% in detecting the number of gallstones via the US. Studies at different time intervals have shown that US has limitations in assessing the number of stones. A study conducted by Brakel et al. [12] found that the accuracy of preoperative ultrasound in determining the number of gallstones was 74%, which is lower than the results reported by Al-Timimi et al. [11].

GB wall thickness assessed via US plays a crucial role in cholecystectomy. However, the US's sensitivity (21%) for detecting thickened GB walls, while specific (100%) and with a high negative predictive value (84%), showed discrepancies compared to intraoperative findings. Some factors like body habitus, bowel gas, and operator experience can affect accuracy. Contrast-enhanced endoscopic ultrasound (EUS) shows promise with a sensitivity of 98% [13]. 

US provided an insight into the condition of the GB (distended/non-distended/ contracted), aiding in surgical planning and anticipating intraoperative challenges. To minimize the complications, a distended GB may be subjected to aspiration before starting dissection. It can be planned preoperatively as per US findings and done under laparoscopic guidance. Our study found significant disparities between the ultrasound and laparoscopic assessments of GB distension. Distended GBs exhibited moderate sensitivity (69.7%) and high positive predictive values (88.6%) on ultrasound, while non-distended GBs showed high specificity (78%) and NPV (91%). Contracted GBs also displayed high specificity (91.7%) and NPV (97.8%). 

Pericholecystic fluid detected by US helps diagnose inflammatory conditions of the GB like cholecystitis (calculous). The presence of inflammatory changes in the target organs may make surgical intervention difficult. Such inflammatory changes detected by US preoperatively can guide the intervention with minimal complications. Pinto et al. conducted a review to study the accuracy of US in the diagnosis of acute calculous cholecystitis. The review mentions a range of sensitivities between 74% and 100%, highlighting the variability of US in detecting pericholecystic fluid [14]. Another study conducted by Wertz et al. reported a sensitivity of 68% for US in detecting pericholecystic fluid in acute cholecystitis patients [15]. In our study, US exhibited perfect sensitivity and NPV (100% for both) with an 83.67% accuracy in detecting pericholecystic fluid.

## Conclusions

Preoperative US findings provide valuable insights for surgical planning. US is highly sensitive and specific for evaluating GB wall thickness and pericholecystic fluid. Preoperative identification of the number of stones, GB distension, and wall thickness correlates well with intraoperative findings, facilitating better surgical outcomes and patient management. Further studies need to be carried out to generalize the results of this study due to the limited sample size. This is attributed to the fact that our study was carried out at a single center over a specific period of time.
